# Ability emotional intelligence profiles and real-life outcomes: a latent profile analysis of a large adult sample

**DOI:** 10.3389/fpsyg.2025.1465774

**Published:** 2025-02-07

**Authors:** Christophe Haag, Lisa Bellinghausen, Clément Poirier

**Affiliations:** ^1^Emlyon, Lyon, France; ^2^Generation QE, Vannes, France; ^3^Qualia Emotion Institute, Lyon, France; ^4^Laboratoire de Psychologie Appliquée et d’Ergonomie, Institut de Psychologie, Université Paris Descartes, Paris, France; ^5^Moodwork, Paris, France

**Keywords:** ability emotional intelligence (AEI), LPA, life outcomes, well - being, decision-making, health, gratitude, emotional intelligence assessment

## Abstract

Few studies have examined emotional intelligence (EI) following a person-centered approach to identify different types of EI profiles and their relationship to everyday life outcomes. Even rarer are those using an “ability” approach of EI (AEI) and related “performance-based” tests, which are considered promising. This study fills this gap by identifying AEI profiles and linking them to everyday outcomes such as health, wellbeing, and decision-making. The QEg (“QE” for Emotional Quotient - Quotient Emotional in French - and “g” for the general population), an ability-based measure of EI, along with other measures, was administered to 2,877 French adults. We then ran latent profile analysis (LPA) and identified three latent profiles within a heterogeneous population. The full emotion processing (FEP) profile outperforms the two others on key domains of life such as stress perception, home-work interaction, gratitude and satisfaction with life, emotional burnout prevention, and decision-making. Our research reveals the need for individualized AEI training programs tailored to three distinct profiles, addressing foundational skills for those with minimal or partial emotional processing while refining existing strengths for those with full emotional processing. Targeting interventions to specific profile characteristics could enhance the effectiveness of AEI training and promote improved wellbeing and life outcomes.

## 1 Introduction

Emotional intelligence (EI) was originally conceptualized by Mayer and Salovey (1997) who defined it as “*the ability to perceive accurately, appraise and express emotion; the ability to access and/or generate feelings when they facilitate thought; the ability to understand emotion and emotional knowledge; and the ability to regulate emotions to promote emotional and intellectual growth*” (p. 10).

Furthermore, EI, now regarded as a promising form of intelligence (Bryan and Mayer, 2021), has given rise to two distinct approaches over time: (a) the “Ability” EI (AEI) model (in line with Mayer and Salovey’s original concept; Haag et al., 2023; Mayer et al., 2004; Schlegel and Mortillaro, 2019) and (b) the “Trait” EI (TEI) model (Bar-On, 1997; Goleman, 1995; Petrides and Furnham, 2000, 2003).

The AEI framework is often seen as more promising compared to TEI as it meets standards of intelligence (Haag et al., 2023, 2024a; Matthews et al., 2004; Mayer et al., 2016; Schlegel and Mortillaro, 2019). While AEI is categorized as a form of intelligence, TEI is regarded as a “personality trait,” linked to conceptual and psychometric challenges (Schlegel and Mortillaro, 2019). Furthermore, research has indicated that these two EI approaches have a weak statistical correlation (Brackett et al., 2006; Haag et al., 2023; Lopes et al., 2004).

The question of whether AEI predicts important real-life outcomes has attracted considerable attention (Robinson, 2024). In such a vein, we explore the association between AEI and real-life outcomes.

### 1.1 The link between AEI and real-life outcomes

Some studies have shown that AEI predicts job performance (O’Boyle et al., 2012), the quality of relationships (Lopes et al., 2004), and overall wellbeing (Di Fabio and Kenny, 2016; Sánchez-Álvarez et al., 2016). However, these associations tend to be relatively weak and inconsistent compared to the predictive power of TEI (Robinson, 2024; Zeidner and Olnick-Shemesh, 2010).

Recently, Robinson (2024) called on researchers to provide fresh insights to know a lot more about whether and how AEI manifests itself in daily life. According to him, AEI should significantly impact decision-making, social interactions, and overall wellbeing, but further research is crucial to “forge” these links. We follow this direction by conducting a latent profile analysis of AEI to identify beneficial and risk profiles in wellbeing, mental and physical health, and decision-making.

### 1.2 The QEg model

Nearly three decades after the publication of their foundational article on AEI (Mayer and Salovey, 1997), Mayer et al. (2016) began to enhance the theoretical framework surrounding the AEI model and its assessment. Following this approach, Haag et al. (2023) introduced the QEPro model, which extends Mayer and Salovey (1997) AEI framework through theory-based item development and scoring method, integrating it within the situational judgment test (SJT) framework. While the QEPro model was specifically developed for managers, the authors later introduced the QEg model, which serves as its generalized version intended for the broader population (Haag et al., 2024a). Both (QEPro and QEg) have the same factorial structure and are composed of the same dimensions. Here, we refer to the QEg model as our target population is a general population of adults.

According to the QEg model, AEI can be defined as the ability to identify (IE), understand (UE), and regulate (SME) emotions (Haag et al., 2024a):

Identification of Emotions (IE) represent the meta-competency to accurately identify emotions in self and others (Haag et al., 2024a). It consists of three abilities:

- Scanning Physiological Manifestations: the ability to recognize one’s own emotions through an introspective examination of the physical sensations felt.
- Interpreting Emotional Cues: the ability to recognize emotions through their cognitive expressions; behavioral inclinations; vocal, postural, and facial cues; along with the related subjective-experiential aspect.
- Identifying Emotional Triggers: the ability to pinpoint the specific triggers of emotions.
  Understanding emotions (UE) represent the meta-competency to accurately understand emotions and anticipate their positive and negative consequences (Haag et al., 2024b). It consists of two abilities:
- Understanding Emotional Timelines: the ability to evaluate the intensity of emotions and to predict how they will change over time.
- Anticipating Emotional Outcomes: the ability to foresee the positive and negative effects of an emotion.
  Strategic Management of Emotions (SME) represents the meta-competency to first select and then feel/express the appropriate emotions to adapt to a situation (Haag et al., 2024b). It consists of two abilities:
- Selecting the Target Emotional State: the ability to recognize and choose the appropriate emotional state for a specific situation.
- Emotion Regulation: the ability to apply the correct emotion regulation strategy to achieve the targeted emotional state.

#### 1.2.1 Measuring emotional intelligence

Conceptualized as a set of abilities, AEI is analogous to general intelligence (Mayer and Salovey, 1997) and should thus be measured through so-called “performance-based” tests - considered the most promising way of assessing EI (Haag et al., 2023; Matthews et al., 2004) but also the most demanding for participants (cognitively and temporally effortful), which explains why few studies use this type of measurement.

New “performance-based” AEI tests emerged recently. Among them, QEg (Haag et al., 2024a), which is an online-only performance-based test that has good psychometric qualities.

The QEg questionnaire aims to measure the AEI seven dimensions of the QEg model defined earlier. The QEg questionnaire extends the framework of the QEPro questionnaire (designed only for managers and leaders) by extracting and revalidating existing items (from QEPro questionnaire), as well as by creating new items relevant to the general public and everyday contexts.

Item development and scoring of the QEg questionnaire are theory-based. Both the items and their “correct” answers are “objectively” determined by following the literature in the field of emotion and emotional intelligence framework (Haag et al., 2024a).

Furthermore, the QEg questionnaire was elaborated within the situational judgment test (SJT) framework to increase criterion-related validity and incremental validity (Lievens et al., 2008).

### 1.3 AEI profiles

Although previous studies have highlighted the key role of emotional intelligence in everyday life, most of them used a variable-centered approach to investigate how individuals differed with regard to the mean of the sample but not how the specific dimensions of emotional intelligence interacted together (Pirsoul et al., 2022).

Moreover, most of these studies used a TEI (rather than AEI) approach (e.g., Martínez-Monteagudo et al., 2021; Pirsoul et al., 2022; Toyama and Mauno, 2016) that considers EI as a “trait” among other personality traits measured through self-report questionnaires (Haag et al., 2023; Matthews et al., 2004; Schlegel and Mortillaro, 2019).

As TEI and everyday life outcomes are commonly measured using self-report questionnaires, these concepts tend to show stronger correlations with each other due to their subjective nature and the consistency in how individuals evaluate their own traits or behaviors (Podsakoff et al., 2003).

In contrast, performance tests, which measure AEI more objectively, often show weaker correlations with self-report scales. This can be attributed to self-assessment biases, where individuals may overestimate or underestimate their actual abilities, leading to discrepancies between self-reported results and objectively measured performance (Kruger and Dunning, 1999; Paulhus and Reid, 1991). Adopting a different perspective to examine the relationship between AEI and life outcomes is thus required.

In this article, we explore AEI following a person-centered approach to identify—through a latent profile analysis (LPA)—AEI profiles regarding QEg’s seven dimensions and an Anova to observe their relationship to everyday life outcomes.

Recently, Haag et al. (2024b) identified two latent profiles of AEI—using the QEPro/QEg model—within a diverse population of managers. LPA, which is specifically structured to account for the presence of subpopulations characterized by different parameters (Meyer and Morin, 2016; Morin, 2016), offers a unique framework for exploring the quantity and nature of emerging AEI profiles.

Their analysis yielded two distinct profiles: The full emotional processing (FEP) and the minimal emotional processing (MEP). The FEP profile significantly outperformed the MEP profile on the following five AEI dimensions: *Identifying Emotional Triggers, Understanding Emotional Timelines, Anticipating Emotional Outcomes, Selecting the Target Emotional State, and Emotion Regulation.* The authors concluded that the FEP displays “the most complete processing of emotion” (p4).

Our approach, in line with Haag et al. (2024b), uses LPA to identify emerging AEI profiles, based on the QEg model and its questionnaire, within a general population, and subsequently examines their associations with real-life outcomes.

## 2 Method

### 2.1 Sample

All participants (*N* = 2,877; Table 1), French native, volunteered in the study without financial incentives and being aware of the confidentiality of their answers. All participants were recruited with the support of a French popular science magazine and a French television program devoted to science which have published on their respective websites a call to participate in our study. They completed QEg and other questionnaires online (via the Qualtrics software package). Test administration was extended over 4 weeks.

**Table 1:** Participants’ socio demographics characteristics.

	Sample
*n*	2,877
Age (years)	33.98
Gender	
Male	959
Female	1918

### 2.2 Measures and procedure

QEg was administered online to all participants. All other measures were French adaptations of self-report questionnaires and were also online administered. We confirmed the psychometric qualities of each of these scales through confirmatory factorial analyses (CFAs) and verification of internal consistency using Cronbach’s alpha. All the CFA results showed a good fit with the presupposed models, and the alpha values were in line with those expected (see Table 2; Nunnally and Bernstein, 1994):

- Perceived Stress Scale 10 (PSS-10; Bellinghausen et al., 2009): χ^2^ (35) = 345.4, CFI = 0.98, RMSEA = 0.056 [0.050, 0.061], TLI = 0.98 et SRMR = 0.045
- Shirom-Melamed Burnout Measure (SMBM; Sassi and Neveu, 2010): χ^2^ (74) = 1216.8, CFI = 96, RMSEA = 0.073 [0.070, 0.077], TLI = 0.96 et SRMR = 0.049
- Satisfaction with Life Scale (SWLS; Bacro et al., 2020): χ^2^ (5) = 48.79, CFI = 0.99, RMSEA = 0.055 [0.042, 0.070], TLI = 0.98 et SRMR = 0.013
- Gratitude Questionnaire-6 (GQ-6; Shankland and Martin-Krumm, 2012): χ^2^ (9) = 134.6, CFI = 0.96, RMSEA = 0.07 [0.060, 0.080], TLI = 0.93 et SRMR = 0.031
- Survey Work–Home Interaction-Nijmegen (SWING; Lourel et al., 2005): χ^2^ (203) =2899.01, CFI = 0.91, RMSEA = 0.068 [0.066, 0.070], TLI = 0.89 et SRMR = 0.057
- Consideration of Future Consequences Scale (CFC-14; Camus et al., 2014): χ^2^ (76) = 818.9, CFI = 0.96, RMSEA = 0.058 [0.055, 0.062], TLI = 0.95 et SRMR = 0.055

**Table 2:** Means and ANOVA results for each variable in function of the three EI profiles.

Variables	*α*	PEP	MEP	FEP	*F*	*η* ^2^
Stress (PSS-10)	0.87	25.4ab	25.85b	24.7a	6.6***	0.0046
Satisfaction with life (SWLS)	0.89	14.58ab	14.14b	15.03a	8.495***	0.0059
Gratitude (GQ-6)	0.74	19.6b	19.21b	20.71a	30.02***	0.0205
Work-home interaction (SWING)						
Work-Home negative interaction	0.88	10.71	10.35	10.63	0.827	
Work-Home positive interaction	0.82	8.63	8.98	8.84	1.463	
Home-Work negative interaction	0.81	3.92b	4.05b	3.54a	6.96***	0.0048
Home-Work positive interaction	0.80	7.99ab	8.37b	7.86a	3.68*	0.0026
Consideration for future consequences (CFC-14)						
Immediate consequences	0.81	18.3b	18.89b	17.40a	15.99***	0.0110
Future consequences	0.75	25.17b	24.89b	26.09a	14.6***	0.0100
Burnout (SMBM)						
Physical fatigue	0.93	24.80	24.22	24.43	0.997	
Emotional exhaustion	0.83	9.25b	9.14b	8.63a	6.144**	0.0042
Cognitive weariness	0.82	17.28	16.85	16.48	2.782	

*** *p* < 0.001; *p < 0.05; PEP, partial emotional processing profile; MEP, minimal emotional processing profile; FEP, full emotional processing profile, letters (a,b,c): differences between groups through pairwise *t*-test.

## 3 Results

### 3.1 Latent profile analysis (LPA)

LPA is designed to determine sub-groups in an extent sample, called a profile. LPA was run with the open-source software Mclust package (Scrucca et al., 2016) to investigate one to five profiles. The optimal number of profiles has been selected in regard to the statistics criterion (see Table 3), substantive meaningfulness, and theoretical conformity (Gillet et al., 2019).

**Table 3:** Parameters of latent profiles analyses.

Classes	AIC	BIC	CAIC	Entropy	BLRT
1	2090	2,173	2,187	1	
2	541	714	743	0.68	1578**
3	32	294	338	0.63	539**
4	−121	230	289	0.65	192.7**
5	−170	271	345	0.64	NA

**p < 0.01; Akaike information criterion (AIC); Bayesian information criterion (BIC); consistent AIC (CAIC); bootstrap likelihood ratio test (BLRT).

**Figure 1:**
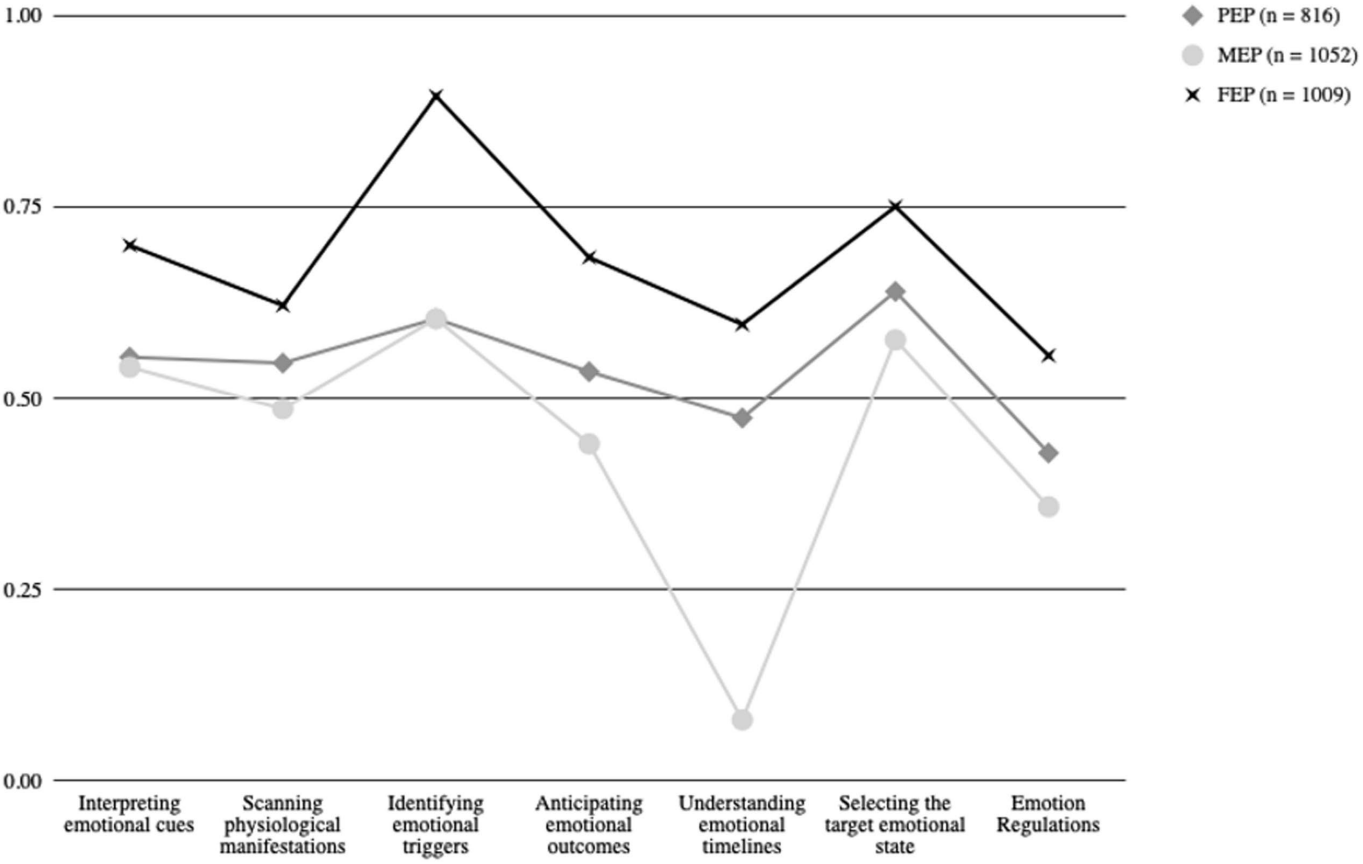
Comparison of three-profile solution based on first-order factor scores.

The three-profile to the five-profile solutions were examined more closely. This examination demonstrated that the three-profile solution was the most proper solution regarding its statistical and theoretical conformity. The three-profile solution was retained for further analyses (BIC = 294; Figure 1).

We named the three profiles full emotional processing (FEP), minimal emotional processing (MEP), and partial emotional processing (PEP). The FEP outperforms the two other profiles on all EI abilities measured by QEg (the subscales appear in Figure 1). These results suggest that the FEP profile captures and processes emotional information in a broader and deeper way than the two other profiles (*Scanning Physiological Manifestations*, *Interpreting Emotional Cues*, *Identifying Emotional Triggers*, *Understanding Emotional Timelines*, *Anticipating Emotional Outcomes*, *Selecting the Target Emotional State*, and *Emotion Regulation*).

We also ran ANOVA in order to understand more deeply the benefits of full emotional processing on real-world outcomes such as perceived stress, home–work interaction, mental health, wellbeing, and decision-making.

### 3.2 FEP’s benefits

We test differences between the three identified profiles (FEP, PEP, and MEP) on several variables through ANOVA. We then compare profiles means two by two with pairwise *t*-test. Our results show that FEP significantly outperforms the two other profiles (MEP performs the worst each time) on all key life variables measured (see Table 2).

FEP is associated with the lowest levels of perceived stress which is consistent with the literature (Shahin, 2020). High perceived stress has a negative impact on mental and physical health (Lu et al., 2019).

FEP is characterized by the lowest score on the home–work negative interaction. Previous research pointed out associations between negative HWI and fatigue (Geurts et al., 2003) and decreased psychological health (Van Hooff et al., 2005).

FEP exhibits and experiences greater gratitude, an emotion that helps individuals to deal with adversity, anxiety, and depression, and to build strong interpersonal relationships (Cunha et al., 2019; Diniz et al., 2023).

FEP has the highest scores on the satisfaction with life scale. Satisfaction with life is associated with better health, higher self-esteem, stronger resilience, lower depression, and anxiety (Diener et al., 2018; Cerezo et al., 2022).

FEP has the lowest score on the Emotional Exhaustion subscale which is considered the core meaning of Burnout (Lee and Ashforth, 1996). Thus, FEP tends to be more protective against depressive symptoms (Gerber et al., 2018).

Finally, our results indicate that FEP has a great consideration of future consequences (vs. immediate gratification) and a tendency to make decisions that are more future-oriented. Not considering the future consequences of our actions is highly risky for our health (Murphy and Dockray, 2018).

The different results suggest that FEP tends to be associated with better mental and physical health, wellbeing, satisfaction with life as well as decision-making.

## 4 Discussion

This article identifies three distinct AEI profiles (FEP, MEP, PEP) using LPA, which expands upon the previous research by Haag et al. (2024b) which identified only two profiles (FEP and MEP) within a (smaller) sample of managers. While both studies used LPA and the QEPro/QEg model framework, the inclusion of a broader, non-managerial adult population in the present research revealed a more nuanced spectrum of AEI profiles.

The emergence of the additional “Partial Emotional Processing” (PEP) profile suggests a greater heterogeneity in emotional intelligence competencies than previously observed within a managerial context. This highlights the importance of considering diverse populations when investigating the relationship between AEI profiles and real-life outcomes.

This article highlights the direct benefits of FEP on various real-life outcomes. Our results show that being a FEP is beneficial for mental and physical health, wellbeing, and decision-making. Consistent with the existing literature, FEP is significantly and negatively associated with high perceived stress, negative work–home interaction, reduced psychological wellbeing, and emotional exhaustion. Conversely, FEP correlated significantly and positively with gratitude, life satisfaction, and the tendency to make decisions that are more future-oriented.

Our findings are in line with Pascual-Leone and Greenberg (2007) who found that distress is often linked to poor or “unprocessed” emotions. They showed, from a therapeutic approach, that there exist more efficient ways of processing emotions than others. Regarding the advantages of FEP on real-life outcomes, FEP could be considered an efficient way of processing emotions.

## 5 Limitations and future research

### 5.1 Participant recruitment bias

While a widely read science magazine and a health-focused television program facilitated the recruitment of a substantial participant pool, this approach may have inadvertently resulted in a sampling bias. Our sampling could bias the generalizability of the findings by disproportionately including individuals who possess a pre-existing interest in science or health-related topics (Franzke et al., 2020). This demographic may limit the generalizability of the study’s conclusions. Future research should consider using more varied recruitment strategies, including outreach to less engaged audiences, to ensure a diverse participant representation that captures a broader spectrum of perspectives on the subject matter (Merriam and Tisdell, 2015).

Not controlling for pre-existing psychiatric and neurological disorders in this study could potentially impact the generalizability and interpretation of the findings. Individuals with such disorders may exhibit unique patterns of emotional processing and regulation that differ substantially from the general population (Aslan et al., 2024; Lezak et al., 2012; Pepping et al., 2024). Failing to account for these differences introduces the risk of confounding variables, obscuring the true relationships between AEI profiles and the measured outcomes. For example, individuals with anxiety disorders may exhibit heightened sensitivity to emotional cues and experience greater emotional reactivity, potentially leading to higher scores on certain QEg dimensions, even in the absence of a “Full Emotional Processing” profile (Barlow, 2002). Conversely, individuals with depressive disorders may show blunted emotional responses, resulting in lower scores regardless of their underlying emotional intelligence competencies (American Psychiatric Association, DSM-5 Task Force, 2013).

This limitation reveals the need for future research incorporating robust screening measures for psychiatric and neurological conditions and potentially implementing stratified analyses to account for their influence on AEI profiles and their associations with real-life outcomes.

### 5.2 Self-report biases

Self-report measures as the ones used in this article to evaluate real-life outcomes, while valuable for assessing subjective experiences, are susceptible to biases such as social desirability response bias and acquiescence bias (Paulhus, 1991; Podsakoff et al., 2003). Future research could incorporate methods to mitigate these biases, such as using multiple informants or using techniques such as the Balanced Inventory of Desirable Responding (BIDR; Paulhus, 1991) to assess response styles. Further, exploring the potential for common method variance through techniques such as Harman’s single-factor test would enhance the robustness of the findings.

*The Need for diverse sampling and methodological approaches.* While LPA provides significant insights into patterns and relationships within data, future research will aim to integrate qualitative data and case studies to enhance the ecological validity of our findings. Incorporating qualitative methods can offer a more profound and nuanced understanding of the contexts and experiences underlying the patterns identified through LPA (Creswell and Poth, 2018). This multi-method approach will allow us to triangulate data sources, thus enriching our interpretations and providing a more comprehensive overview of the phenomena under investigation (Flick, 2018). By leveraging both quantitative and qualitative techniques, we aspire to refine our findings and ensure that they are not only statistically robust but also grounded in real-world experiences and perspectives (Yin, 2018).

### 5.3 Examining specific cognitive and emotional regulation strategies

As LPA shows, emotion regulation considered as meta-competence (Peña-Sarrionandia et al., 2015) is high when the scores on the six dimensions preceding “emotion regulation” are high too. This first insight encourages us in the future to examine if FEP is a “cascading” profile (Joseph and Newman, 2010). Future research could examine the specific cognitive and emotional regulation strategies used by individuals with the FEP profile and explore if a cascading model emerges. This could involve measures of cognitive appraisal, coping mechanisms, and different types of emotion regulation strategies (Gross and Ford, 2024).

*Exploring contextual factors*. Future research could explore the factors contributing to the emergence of the PEP profile, potentially focusing on contextual factors such as work environment, age, or personality traits. Such a comparative analysis underscores the utility of person-centered approaches for uncovering a more comprehensive understanding of the multifaceted nature of AEI and its impact on various life domains.

## 6 Implications

The identification of distinct AEI profiles necessitates a shift from generic to personalized AEI training programs. A “one-size-fits-all” approach is demonstrably insufficient given the inherent heterogeneity of emotional processing styles, as evidenced by this study and prior research (Gross and Ford, 2024; Joseph and Newman, 2010).

Individuals exhibiting the *full emotional processing* (FEP) profile, characterized by robust emotional competence across all dimensions of the QEg model (Haag et al., 2024a), would benefit from advanced training focusing on the refinement and extension of existing strengths. Interventions might emphasize advanced emotion regulation strategies within complex scenarios, cultivating emotional leadership skills, and leveraging their emotional intelligence to enhance decision-making in high-stakes situations.

Conversely, individuals classified as *minimal emotional processing* (MEP) exhibit substantial deficits across multiple facets of emotional processing. Therefore, training should prioritize foundational skill-building, beginning with the development of emotional literacy. This involves enhancing the ability to accurately identify and label emotions in self and others, fostering comprehension of the causes and consequences of emotional experiences, and introducing fundamental emotion regulation techniques such as antecedent-focused regulation as well as cognitive reappraisal (Gross and Ford, 2024). The observation and understanding of one’s actual emotional functioning will precede the utilization of role-playing exercises and case studies for optimal learning and skill development, acquisition, and application (Greenberg, 2020).

The *partial emotional processing* (PEP) profile reveals a more nuanced presentation, with varying levels of proficiency across different dimensions of the QEg model. Consequently, interventions must adopt a highly targeted and adaptive approach. A comprehensive assessment of each individual’s specific strengths and weaknesses is crucial in designing personalized training plans that directly address identified areas of deficiency while simultaneously building upon existing competencies. This requires a precise mapping of skills gaps onto the QEg dimensions (Haag et al., 2024a) to inform the development of individualized training strategies.

Regardless of the EI profile, continuous feedback is crucial for optimal progress. This should incorporate multifaceted feedback mechanisms, including self-monitoring tools, peer feedback sessions, and coach-led assessments to provide comprehensive and iterative learning.

The utilization of situational judgment tests (SJTs), reflecting the QEg’s foundation, enables effective training by simulating real-world scenarios in which participants could practice applying emotional processing skills within realistic contexts.

Finally, the adoption of a mixed-methods approach, incorporating both quantitative and qualitative data (interviews, focus groups), is essential for a more nuanced understanding of the training’s effectiveness and for facilitating program refinement and adaptation.

## Data Availability

The datasets presented in this study can be found in online repositories. The names of the repository/repositories and accession number(s) can be found at: https://osf.io/phq5y/?view_only=56cb8431148d442894d5a330296ce094.
